# Modeling human endothelial cell transformation in vascular neoplasias

**DOI:** 10.1242/dmm.012674

**Published:** 2013-09

**Authors:** Victoria W. Wen, Karen L. MacKenzie

**Affiliations:** 1 Cancer Cell Development Group, Children’s Cancer Institute Australia for Medical Research, Lowy Cancer Research Centre, University of New South Wales, Randwick, NSW, Australia

## Abstract

Endothelial cell (EC)-derived neoplasias range from benign hemangioma to aggressive metastatic angiosarcoma, which responds poorly to current treatments and has a very high mortality rate. The development of treatments that are more effective for these disorders will be expedited by insight into the processes that promote abnormal proliferation and malignant transformation of human ECs. The study of primary endothelial malignancy has been limited by the rarity of the disease; however, there is potential for carefully characterized EC lines and animal models to play a central role in the discovery, development and testing of molecular targeted therapies for vascular neoplasias. This review describes molecular alterations that have been identified in EC-derived neoplasias, as well as the processes that underpin the immortalization and tumorigenic conversion of ECs. Human EC lines, established through the introduction of defined genetic elements or by culture of primary tumor tissue, are catalogued and discussed in relation to their relevance as models of vascular neoplasia.

## Introduction

Vascular neoplasias are a spectrum of disorders that include rare, aggressive malignancies such as angiosarcoma, as well as commonly occurring infantile hemangiomas that are usually indolent but can give rise to life-threatening complications when vital organs are involved (Drolet et al., 1999; Szajerka and Jablecki, 2007; Koch et al., 2008; Young et al., 2010). The unifying pathological feature of these conditions is perturbation of endothelial cell (EC) proliferation and disorganization of endothelial tissue architecture. In a non-pathological state, mature ECs have limited proliferative ability and form an organized monolayer along the inner surface of blood and lymphatic vessels. These cells are derived from a hierarchy of highly proliferative progenitors that are thought to reside within the bone marrow and are ontologically related to hematopoietic stem cells (Asahara et al., 1997; Asahara et al., 1999). An array of cytokines and chemokines direct the recruitment and homing of endothelial progenitors from the bone marrow, via the peripheral circulation, to the inner walls of blood and lymph vessels where they differentiate and contribute to many normal physiological processes, such as wound healing, inflammation, coagulation, cell migration and hematopoiesis (Shi et al., 1998; Asahara et al., 1999; Krishnaswamy et al., 1999; Takahashi et al., 1999).

The mitogenic signaling pathways that regulate the proliferation of normal ECs have been intensively studied and are reviewed elsewhere (Carmeliet and Jain, 2011). However, relatively few investigations have focused on the molecular mechanisms that promote unregulated proliferation and transformation of ECs during the development of vascular neoplasia. This is in part due to the rarity of EC-derived malignancies as well as the focus of most investigations on end-stage disease, which precludes study of the molecular events involved in the initiation and progression of disease. Consequently, little progress has been made in the development of novel therapeutics for the treatment of endothelial malignancies over the past few decades. By describing the molecular features of vascular neoplasias, as well as the cell lines and *in vivo* models that are available to model EC transformation, the aim of this review is to inform and motivate preclinical studies of new therapeutic approaches to the treatment of specific EC neoplasias.

## Clinical and molecular features of EC-derived neoplasias

### Hemangioma

Hemangioma is the most common tumor of infancy, affecting ∼10% of Caucasian neonates (Table 1) (Drolet et al., 1999). This neoplasia develops as a rapidly growing disorganized mass of ECs. Most hemangiomas appear within the first few weeks of birth and are characterized by an initial period of rapid tumor growth that is driven by the proliferation of immature ECs. This is followed by a ‘rest phase’, in which there is little change in the appearance of the tumor. A subsequent slow involuting phase associated with EC differentiation and apoptosis marks regression of the tumor in almost all cases. The involuted tumor is replaced by fibrous and fatty tissue. Although histologically benign, non-invasive and unlikely to progress to a malignancy, visceral hemangiomas can result in substantial morbidity and mortality by causing obstruction, hemorrhage or by diverting blood flow. Currently available treatments for life-threatening or otherwise complicated cases of hemangioma have limited efficacy and commonly result in treatment-associated toxicity.

**Table 1.**
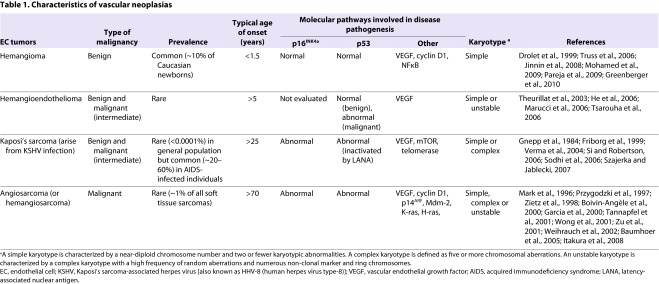
Characteristics of vascular neoplasias

Hemangiomas are composed of morphologically normal, immature ECs that are clonally derived and exhibit enhanced proliferation and migration (Mulliken et al., 1982; Boye et al., 2001; Dadras et al., 2004). Evidence has recently emerged to suggest that the excessive proliferation of ECs in hemangiomas is driven by an imbalance in angiogenic signaling factors and activation of nuclear factor-κB (NFκB) (Jinnin et al., 2008; Greenberger et al., 2010). Notably, a recent study of genetic risk factors for hemangioma identified germline mutations in the genes encoding vascular endothelial growth factor (VEGF) receptor 2 (VEGFR2) and anthrax toxin receptor 1 (also known as tumor endothelial marker 8) that perturb VEGF signaling and contribute to disorganized angiogenesis (Jinnin et al., 2008). Other molecular events resulting from the cytogenetic abnormalities that have been identified in hemangiomas could also play a role in this disorder. Chromosomal aberrations that have been observed in hemangioma include loss of heterozygosity at chromosome regions 5q, 13q14 and 17p13, as well as loss of the Y chromosome and amplification of the cyclin D1 gene on chromosome 11 (Berg et al., 2001; Domfeh et al., 2006; Mohamed et al., 2009). Insights into the molecular regulation of different phases of hemangioma will be valuable for the development of improved therapies for these disorders.

### Intermediate grade vascular malignancies

**Fig. 1.**
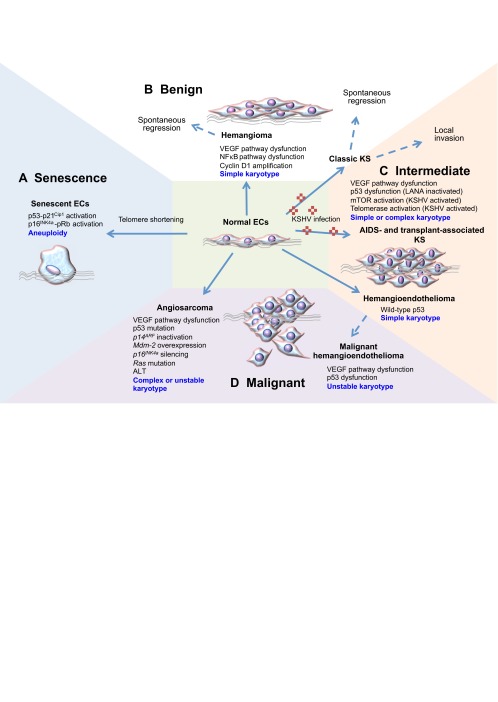
**Molecular alterations underlying vascular neoplasias.** (A) Normal ECs (illustrated in the center) have a finite replicative lifespan that is dictated by the shortening of telomeres with each cell division. Critical shortening of telomeres leads to activation of p53 and pRb pathways, and the onset of senescence. (B–D) Abnormal proliferation of ECs is a common feature of vascular neoplasias. Abnormalities in the VEGF pathway are common across the spectrum of vascular neoplasias. Other mechanisms implicated in oncogenesis are listed, and chromosomal characteristics are described in blue. A simple karyotype is characterized by a near-diploid chromosome number and two or fewer karyotypic abnormalities. A complex karyotype is defined as five or more chromosomal aberrations. (B) Hemangioma is a benign condition in which hyperproliferation of immature ECs is driven by constitutive activation of NFκB and VEGF signaling pathways. (B,C) Infection by KSHV gives rise to classic Kaposi’s sarcoma (KS), which is relatively indolent and can either undergo spontaneous regression or can become locally invasive. p53 is inactivated, whereas mTOR and telomerase are activated by the KSHV-encoded protein LANA and the G-protein coupled receptor, respectively. (C) In immune-compromised individuals, KSHV often progresses to a multifocal tumor. (C,D) Hemangioendothelioma also presents in various grades, with defects in the p53 pathway found in malignant hemangioendothelioma, but not in intermediate-grade hemangioendothelioma. Chromosomal abnormalities are observed in intermediate-grade and malignant EC tumors. (D) In addition to p53 dysfunction, malignant hemangioendothelioma and angiosarcoma often exhibit chromosomal instability. Activation of oncogenes such as *Ras* is also common in angiosarcoma. EC, endothelial cell; NFκB, nuclear factor-κB; ALT, alternate telomere lengthening; KS, Kaposi’s sarcoma; KSHV, Kaposi’s sarcoma-associated herpes virus [also known as HHV-8 (human herpes virus type-8)]; VEGF, vascular endothelial growth factor; LANA, latency-associated nuclear antigen; AIDS, acquired immune deficiency syndrome; pRb, retinoblastoma tumor suppressor protein; mTOR, mammalian target of rapamycin.

Hemangioendotheliomas and Kaposi’s sarcoma (KS) are intermediate-grade endothelial tumors that are ranked between benign hemangioma and aggressive angiosarcoma in terms of malignancy (Table 1; Fig. 1) (Weiss and Enzinger, 1986; Salahuddin et al., 1988; Mentzel et al., 1997). Hemangioendotheliomas are rare tumors of variable and unpredictable malignancy that have been diagnosed in adults of most age groups. These neoplasms most often present as low-grade tumors in the trunk or limbs (Weiss and Enzinger, 1986; Mentzel et al., 1997). The lesions arise from blood vessels and are composed of cells that have been phenotypically identified as vascular ECs (Mentzel et al., 1997; Pyakurel et al., 2006). Approximately 15% of patients die of disease associated with distant metastases (Mentzel et al., 1997). The molecular biology of hemangioendothelioma is largely uncharacterized, although VEGF expression and abnormalities in the p53 tumor suppressor pathway have been shown to be associated with less-differentiated (defined by a lack of endothelial-specific storage granules known as Weibel-Palade bodies) tumor regions in a patient with metastatic disease (Theurillat et al., 2003). Chromosomal instability has been reported in malignant hemangioendothelioma (Theurillat et al., 2003; Tsarouha et al., 2006).

KS encompasses a group of neoplasms in which the lesions are comprised of proliferating ‘spindle cells’ of endothelial origin (Salahuddin et al., 1988; Kaaya et al., 1995; Ganem, 2010). Classic KS occurs predominantly in elderly men, although endemic subtypes are observed among younger adults and children in specific geographical regions of Africa (Szajerka and Jablecki, 2007). Classic KS is a relatively indolent disease that is rarely life-threatening and is often left untreated. Indeed, spontaneous remissions have been reported (Brooks, 1986). Cases of KS that do progress usually involve local invasion, whereas distant dissemination is extremely rare. KS also manifests in association with immune suppression in individuals with AIDS and organ-transplant recipients (Gnepp et al., 1984). In contrast to classic KS, AIDS-associated KS presents as a more widespread, multifocal disorder where life-threatening complications can result from visceral involvement.

KS arises in individuals infected with human herpes virus 8 [also known as KS-associated herpes virus (KSHV)] (Chang et al., 1994). Since the discovery of KSHV, there has been considerable investigation of the genetics and oncogenic potential of this virus (reviewed by Ganem, 2010). The KSHV genome encodes viral homologs of cellular genes that promote the cell cycle and cellular immortalization, inhibit apoptosis, stimulate angiogenesis, and enable infected cells to evade the immune system (Boshoff et al., 1995; Bais et al., 1998; Knight et al., 2001; Bais et al., 2003; Sodhi et al., 2006; Chang et al., 2009). Consequently, activation of cellular oncogenes and mutation of tumor suppressor genes might play a less substantial role in the initiation of KS relative to other sarcomas. Although *TP53* (encoding p53) mutations seem to be rare in KS, p53 protein function can be suppressed through direct interaction with the KSHV-encoded latency-associated nuclear antigen (LANA) (Friborg et al., 1999). Insights into the role of KSHV in the pathogenesis of KS have provided a platform for several early phase clinical trials of new therapeutic strategies, which pave the way for rapid progress towards the more effective treatment of AIDS- and transplant-related KS. Promising novel therapeutics include inhibitors of tyrosine kinase signaling and compounds that target the mTOR (mammalian target of rapamycin) pathway, which are activated by the KSHV G-protein coupled receptor (Koon et al., 2005; Sodhi et al., 2006).

### Angiosarcoma

Angiosarcoma is a rare malignancy that makes up less than 1% of all sarcomas. In contrast to other EC-derived neoplasias, angiosarcomas are highly malignant (Table 1; Fig. 1) (Young et al., 2010). This malignancy occurs with a peak incidence in the seventh decade of life and tends to arise in the viscera, skin and soft tissue. Angiosarcomas have a propensity to recur locally and spread widely, exhibiting a high rate of lymph node and systemic metastases. The disease is associated with a high mortality rate as a result of poor response to treatment (Mark et al., 1996; Lezama-del Valle et al., 1998). With a reported 5-year survival rate of ∼20% and very little change in treatment options over the past decades, innovative therapies are urgently needed. Tumor resection with adjuvant radiotherapy is currently the main treatment approach to angiosarcoma, although a few groups have recently begun to pursue biological and molecular targeted approaches (Koontz et al., 2008; George et al., 2009; Maki et al., 2009; Young et al., 2010). A more detailed understanding of the molecular pathways involved in malignant transformation of ECs and more extensive preclinical studies will be invaluable for the validation of these therapies and the optimization of effective therapeutic strategies.

Most cases of angiosarcoma arise sporadically; however, previous irradiation, exposure to toxic chemicals and chronic lymphedema are known risk factors (Mark et al., 1996; Lezama-del Valle et al., 1998). Activation of the oncogene *K-ras* through acquisition of specific point mutations is a common event in angiosarcoma, reportedly occurring in 29% of hepatic angiosarcomas (7 out of 24) and 60% of cardiac angiosarcomas (3 out of 5) (Przygodzki et al., 1997; Boivin-Angèle et al., 2000; Garcia et al., 2000). The incidence of K-ras mutations seems to be highest among angiosarcomas associated with exposure to vinyl chloride, where mutations have been detected in ∼80% of cases (5 out of 6 cases tested) (Boivin-Angèle et al., 2000). Ras mutations are likely to contribute to the highly metastatic nature of angiosarcomas, given that they are generally not found in non-malignant vascular neoplasias (Table 1). The malignant growth of angiosarcoma is driven by the proliferation of pleomorphic cells that exhibit varying degrees of EC differentiation and give rise to a chaotic tissue architecture (Young et al., 2010). VEGF is expressed at high levels in angiosarcoma and the expression of VEGF receptors is associated with a more differentiated tumor phenotype and favorable prognosis (Zietz et al., 1998; Itakura et al., 2008). Consistent with those observations, inhibition of VEGF receptor 1 (VEGFR1) has been shown to increase the proliferation of canine angiosarcoma cells *in vitro* (Tamburini et al., 2009).

Abnormalities in the *TP53* pathway – including p53 mutations, Mdm-2 overexpression and inactivation of p14^ARF^ – are common molecular aberrations in angiosarcoma (Hollstein et al., 1994; Zietz et al., 1998; Weihrauch et al., 2002). For example, mutations in the *TP53* gene were detected in ∼30% of liver angiosarcomas (Przygodzki et al., 1997; Weihrauch et al., 2002). Consistent with the high frequency of p53 dysfunction, complex karyotypes and chromosomal instability are often observed in angiosarcomas. Chromosomal aberrations reported in angiosarcomas include deletions within the *CDKN2A* locus at chromosome region 9p21, which encodes tumor suppressors p16^INK4a^, p15^INK4b^ and p14^ARF^. Inactivation of p16^INK4a^ by promoter methylation is another frequent event in liver angiosarcoma (Tannapfel et al., 2001; Weihrauch et al., 2002). Studies of liver angiosarcoma showed that p16^INK4a^ was repressed by promoter methylation in 63% (12 out of 19) of tumor samples, whereas 5% (1 out of 19) exhibited homozygous deletion and 10% (2 of 19) exhibited loss of heterozygosity at the *CDKN2A* locus. Overall, inactivation of p16^INK4a^ was detected in 74% (14 out of 19) of angiosarcomas. The importance of p16^INK4a^ repression in angiosarcoma was also evident from canine studies that showed sustained expression of p16^INK4a^ in 100% of benign hemangiomas (a total of ten were examined), and p16^INK4a^ suppression in 82% (32 out of 39) angiosarcomas (Yonemaru et al., 2007). Together, these studies implicate Ras activation plus aberrations in the p53 pathway and silencing of p16^INK4a^ as key events in the pathogenesis of advanced, but not benign, vascular tumors (Table 1, Fig. 1).

## Immortalization and tumorigenic conversion of human ECs

### The limited replicative potential of normal human ECs

A fundamental step in the development of endothelial neoplasias is the deregulation of cell proliferation. Normal human cells have a finite replicative ability, yet cancer cells are capable of unlimited replication, i.e. they are immortal. Cellular immortality is considered to be a requirement for malignant transformation and is a defining property of the subset of tumor cells that drive tumor growth and recurrence (Zhou et al., 2009). Previous studies have revealed that the *in vitro* replicative potential of normal human cells, including ECs, is restricted by both intrinsically and extrinsically initiated stresses that induce senescence (e.g. telomere shortening and an oxidative environment, respectively) (Hayflick and Moorhead, 1961; Johnson and Longenecker, 1982; Yuan et al., 2008). Senescent ECs typically adopt a flattened morphology and demonstrate cytoplasmic spreading, increased granularity, vacuolization and multi-nucleation. They are resistant to mitogenic stimulation and exhibit pH-dependent β-galactosidase activity (Johnson and Longenecker, 1982; Dimri et al., 1995).

It is well established that the shortening of chromosomal-end structures, referred to as telomeres, plays a central role in the onset of senescence (Artandi and DePinho, 2010). Telomeres normally function to prevent chromosomal end-to-end fusions and to maintain genomic integrity during cell division. However, telomeric DNA is eroded during consecutive cell divisions as a consequence of the inability of DNA polymerases to synthesize the 5′ terminus of linear DNA. Exposure to oxidizing agents accelerates telomere shortening and the onset of EC senescence; conversely, free radical scavengers reduce the rate of telomere shortening (von Zglinicki et al., 1995; Xu et al., 2000; Kurz et al., 2004; Napier et al., 2010). The progressive shortening of telomeres has been demonstrated in serially passaged cultures of human ECs derived from the umbilical vein, iliac arteries and veins, abdominal aorta, and bone marrow (Chang and Harley, 1995; Aviv et al., 2001; MacKenzie et al., 2002). Telomere shortening has also been demonstrated in arterial ECs *in vivo* in association with increasing age (Okuda et al., 2000).

When telomeres reach a critically short length, they are prone to fusion and promote chromosome rearrangements. Hence, primary ECs that are maintained in culture for long periods of time frequently harbor aberrant chromosomes (Wagner et al., 2001). In addition to structural rearrangements, changes in whole chromosome number (aneuploidy) tend to occur as cultured ECs age and approach senescence *in vitro* (Nichols et al., 1987; Johnson et al., 1992; Zhang et al., 2000; Aviv et al., 2001). In normal human cells, chromosome fusions and rearrangements initiate a DNA damage response that culminates in activation of tumor suppressor p53 and transcriptional upregulation of p21^Cip1/Waf1^, which halts cell cycle progression and initiates senescence (Stein et al., 1999). Tumor suppressor p16^INK4a^ is subsequently upregulated, resulting in hypo-phosphorylation of the retinoblastoma tumor suppressor protein (pRb) and a sustained growth arrest (Munro et al., 1999; Wagner et al., 2001; Freedman, 2005). The activation of tumor suppressor pathways and induction of senescence plays a crucial role in preventing replication of aged endothelial cells harboring cytogenetic aberrations. Disruption of these mechanisms enables cells to continue to proliferate and initiate neoplastic growth, as outlined below.

### Lifespan extension, immortality and genetic evolution of ECs

During neoplastic transformation, the replicative lifespan of human cells is extended by either the activation of a telomere maintenance mechanism (described below) or inactivation of a tumor suppressor pathway (reviewed by Reddel, 2000). Inactivation of tumor suppressor pathways enables cells to progress through the cell cycle with critically short telomeres. However, short, dysfunctional telomeres that are present in proliferating cells promote chromosomal fusions and genomic instability (Ray et al., 1990; Counter et al., 1992). Telomere dysfunction in pre-malignant and cancerous cells is typically evidenced by dicentric chromosomes, unbalanced translocations, gene amplifications, deletions and ring chromosomes (Ducray et al., 1999; Gisselsson et al., 2001; O’Hagan et al., 2002). These types of chromosomal aberrations are frequently observed in KS and angiosarcoma, and have also been identified in immortalized EC lines, particularly those with defective tumor suppressor pathways (Schuborg et al., 1998; Wong et al., 2001; Wen et al., 2006). Chromosomal instability accelerates the rate and accumulation of molecular changes that facilitate malignant transformation and tumor progression (Rudolph et al., 2001), including activation of a telomere maintenance mechanism, oncogene activation or silencing of tumor suppressor genes.

Under standard culture conditions, ECs that bypass senescence usually succumb to a proliferative ‘crisis’ that is characterized by an increased rate of cell death and reduced cell expansion (MacKenzie et al., 2002; Gu et al., 2003; Nisato et al., 2004; Wen et al., 2006). Crisis is thought to be initiated by telomere dysfunction, genetic catastrophe and/or fatal oxidative damage. Cells that are driven into crisis by the deactivation of tumor suppressor pathways resume proliferation if they undergo a spontaneous molecular event that activates a telomere maintenance mechanism: either activation of the enzyme telomerase or an alternate telomere lengthening mechanism (ALT) (Counter et al., 1992; Bryan et al., 1995). Telomere maintenance stabilizes chromosomes and thereby enables both escape from crisis and unlimited proliferation.

### Extension of replicative lifespan and immortalization of ECs following transfection with viral antigens

The extended replicative lifespan that results from the inactivation of tumor suppressor pathways is thought to represent a pre-malignant stage of the multistep process of cancer development. Several investigations have demonstrated that the replicative lifespan of normal human ECs derived from bone marrow microvasculature, the umbilical vein and the iliac vein is extended by transfection with Simian virus 40 (SV40) T antigen or human papilloma virus (HPV)-encoded E6 and E7 oncogenes (Table 2). The large T antigen of SV40 and the HPV E6 and E7 oncogenes functionally inactivate the p53 and pRb tumor suppressor pathways and thereby enable transfected cells to bypass senescence (Girardi et al., 1965; Werness et al., 1990; Shay et al., 1991). Viral-antigen-transfected ECs have been shown to replicate for up to 42 passages beyond senescence (Table 2) (Gimbrone and Fareed, 1976; Schwartz et al., 1991; Fickling et al., 1992; Hohenwarter et al., 1992b; Lassalle et al., 1992; Bailey et al., 1994; Rhim et al., 1998; Krump-Konvalinkova et al., 2001; O’Hare et al., 2001; MacKenzie et al., 2002).

**Table 2.**
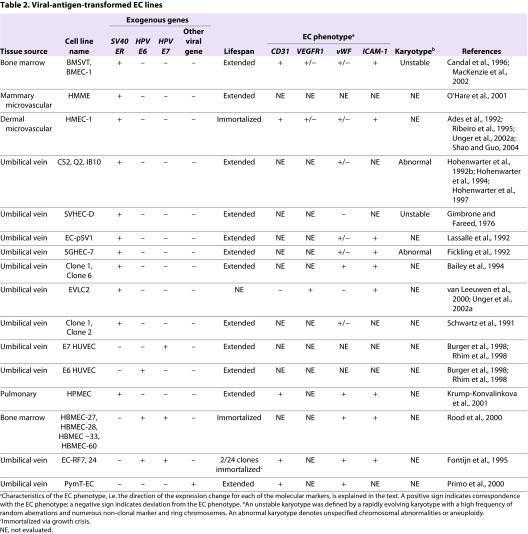
Viral-antigen-transformed EC lines

A number of studies also reported that viral transformation leads to immortalization of ECs (Ades et al., 1992; Fontijn et al., 1995; Burger et al., 1998; Rhim et al., 1998; Rood et al., 2000). However, a growth crisis prior to immortalization was noted in several studies (Ades et al., 1992; Fontijn et al., 1995; Rood et al., 2000; MacKenzie et al., 2002) and two studies showed that telomerase was spontaneously activated in the immortalized ECs (Rhim et al., 1998; Gagnon et al., 2002). Ectopic activation of telomerase was shown to cooperate with SV40 transformation to overcome crisis and efficiently promote immortalization of bone-marrow- and mammary-derived ECs (O’Hare et al., 2001; MacKenzie et al., 2002). Together, these studies suggest that inactivation of tumor suppressor pathways and activation of a telomere maintenance mechanism is necessary and sufficient for *in vitro* immortalization of human ECs.

Immortalization of ECs by transfection with viral oncogenes and activation of telomerase is not sufficient for malignant conversion of ECs (Burger et al., 1998; Rhim et al., 1998; MacKenzie et al., 2002). Nevertheless, viral-antigen-transfected ECs do exhibit some characteristics of transformed cells, including the ability to proliferate independently of exogenous VEGF, and reduced expression of proteins that define the endothelial phenotype, including VEGFR2, platelet endothelial cell adhesion molecular-1 (CD31; also known as PECAM-1), VE cadherin and von Willebrand factor (vWF) (Table 2) (Gimbrone and Fareed, 1976; Hohenwarter et al., 1992b; Rood et al., 2000; MacKenzie et al., 2002; Shao and Guo, 2004). These features parallel the abnormal expression of VEGF receptors and tendency for downregulation of vWF and CD31 in late-stage hemangiomas (Takahashi et al., 1994). Viral-oncogene-transformed ECs also accumulate genomic rearrangements that are similar to those observed in EC malignancies (Gimbrone and Fareed, 1976; MacKenzie et al., 2002).

### Telomerase-mediated immortalization of human ECs

The active core of the telomerase holoenzyme is a ribonuclear protein complex that includes a reverse transcriptase (hTERT), a non-coding RNA that functions as a template for telomere synthesis (hTR) and an RNA-binding and -modifying protein, dyskerin, which stabilizes the RNA component (Feng et al., 1995; Nakamura et al., 1997; Cohen et al., 2007). Although telomerase is not expressed in mature somatic cells, its activation accounts for telomere maintenance in ∼85% of all human cancers and immortal cell lines. Telomerase activity is also readily detected in human germ cells, stem cells and normal endothelial progenitors (Kim et al., 1994; Wright et al., 1996). Telomerase is downregulated during development, differentiation and with *in vitro* passage (Hsiao et al., 1997; Ingram et al., 2005). Consequently, it is not usually detectable in differentiated ECs.

Ectopic expression of hTERT reactivates telomerase in mature ECs and is further bolstered by co-expression of hTR (Yang et al., 1999; MacKenzie et al., 2002; Gu et al., 2003; Nisato et al., 2004; Napier et al., 2010). Reactivation of telomerase activity has been shown to extend the replicative lifespan of normal human ECs, but is not sufficient for immortalization. A number of studies have shown that ECs that overexpress hTERT are subject to a growth crisis following an extended period of replication (Krump-Konvalinkova et al., 2001; O’Hare et al., 2001; MacKenzie et al., 2002; Gu et al., 2003; Nisato et al., 2004; Wen et al., 2006; Takano et al., 2008) (Table 3). The occurrence of crisis in hTERT-transduced EC cultures highlights a requirement for additional molecular events to cooperate with telomerase in the immortalization process. The efficient immortalization of ECs co-transfected with hTERT and viral oncogenes is consistent with the inactivation of tumor suppressor genes being a crucial requirement (Table 3) (O’Hare et al., 2001; MacKenzie et al., 2002). This is further supported by studies that showed that hTERT-transduced ECs frequently silenced tumor suppressor p16^INK4a^, which functions as a major regulator of the RB pathway during hTERT-driven immortalization (Wen et al., 2006). Furthermore, abnormalities in the *TP53* pathway have also been reported in hTERT-immortalized EC lines (Wen et al., 2006). This inactivation of tumor suppressor pathways is likely to be required for telomerase-transduced ECs to overcome cellular stress (e.g. oxidative stress) during immortalization.

**Table 3.**
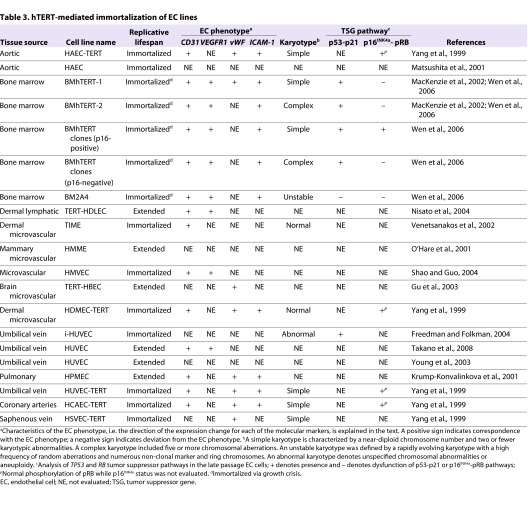
hTERT-mediated immortalization of EC lines

EC lines immortalized following hTERT transduction retain many of the properties of normal human ECs, including continued expression of normal EC surface markers, including CD31 and ICAM-1, and a requirement for VEGF signaling for continued proliferation (Table 3) (Yang et al., 1999; Krump-Konvalinkova et al., 2001; MacKenzie et al., 2002; Nisato et al., 2004; Shao and Guo, 2004; Takano et al., 2008; Dill et al., 2012). Interestingly, some hTERT-transduced EC lines were also shown to form functional vessels *in vivo* and *in vitro* (Yang et al., 2001; MacKenzie et al., 2002; Freedman and Folkman, 2004). Thus, despite their deregulated replicative lifespan, hTERT-immortalized ECs generally seem to retain more accurately the properties of normal, untransformed ECs than do viral-antigen-transformed ECs. Nevertheless, it is important to note that spontaneous molecular alterations that occur during hTERT-driven immortalization can confer neoplastic properties. For instance, our recent investigations revealed that repression of p16^INK4a^ during immortalization of ECs is associated with cytoskeletal changes, altered motility and defects in morphogenesis *in vitro* (Kan et al., 2012). These changes are likely to reflect some of the phenotypic changes required for the acquisition of metastatic ability *in vivo*.

Repression of p16^INK4a^ by promoter methylation in hTERT-immortalized ECs is a molecular event that has also been observed in angiosarcoma (Fig. 1) (Tannapfel et al., 2001; Weihrauch et al., 2002). In our study of hTERT-transduced bone marrow ECs (BMECs), the subset of clones that repressed p16^INK4a^ all developed complex karyotypes (Wen et al., 2006). The cytogenetic heterogeneity and abnormalities detected in the p16^INK4a^-negative BMECs were typical of those that have been documented in EC-derived tumors. Specific aberrations that recur in p16^INK4a^-repressed cell lines and are also observed in angiosarcoma include monosomy 13, loss of the Y chromosome, rearrangements involving 17p, and trisomies 20 and 11 (Mandahl et al., 1990; Kindblom et al., 1991; Gil-Benso et al., 1994; Cerilli et al., 1998; Schuborg et al., 1998; Wong et al., 2001; Zu et al., 2001; Baumhoer et al., 2005; Tsarouha et al., 2006; Wen et al., 2006). Repression of p16^INK4a^ and the concurrent development of karyotypic complexity is a feasible depiction of the molecular alterations that permit the accumulation of genomic imbalances in EC neoplasias.

Relatively little is known about the mechanism(s) that support telomere maintenance in the various types of human vascular neoplasias in patients. However, one study demonstrated telomerase activity in 22 out of 22 KS lesions (Chen et al., 2001), and others have shown that KSHV-encoded LANA directly activates Sp1-mediated transcription of hTERT (Knight et al., 2001; Verma et al., 2004). In contrast, an assessment of telomerase activity in canine angiosarcomas showed no detectable telomerase enzyme activity in 6 out of 7 cases (Yazawa et al., 1999; Chandler et al., 2009). Telomerase activity was also undetectable in two single-clone-derived angiosarcoma cell lines, ISO-HAS.1 and AS-M.5 (Krump-Konvalinkova et al., 2003). These studies suggest that ALT could be a common mechanism of telomere length maintenance in angiosarcoma. However, this possibility remains to be confirmed by demonstration of the specific molecular characteristics of ALT-immortalized cells, such as very long heterogeneous telomere lengths, DNA c-circles and PML bodies in angiosarcoma tissue (Bryan et al., 1997; Henson et al., 2005; Fasching et al., 2007).

### Tumorigenic conversion of human EC lines

Immortalized EC lines established by co-expression of hTERT and SV40 T antigen exhibit a partially transformed phenotype (Table 4) (Salmon et al., 2000; O’Hare et al., 2001; MacKenzie et al., 2002; Krump-Konvalinkova et al., 2003). BMECs that co-expressed SV40 T antigen and hTERT were shown to be capable of robust anchorage-independent growth in soft-agarose, but were not overtly tumorigenic, because implantation in immune-compromised mice resulted in very small subcutaneous nodules that regressed within 3 weeks (MacKenzie et al., 2002). Pulmonary and sinusoidal ECs transformed with hTERT and SV40 ER were similarly non-tumorigenic (Salmon et al., 2000; Krump-Konvalinkova et al., 2001). In addition to the downregulation of phenotypic markers, ECs immortalized by SV40 T antigen grew independently of exogenous VEGF (Tables 2, 4), which is consistent with the constitutive activation of VEGF signaling and heterogeneous phenotype observed in human vascular neoplasias (Ohsawa et al., 1995; MacKenzie et al., 2002; Bais et al., 2003; Itakura et al., 2008; Young et al., 2010).

**Table 4.**
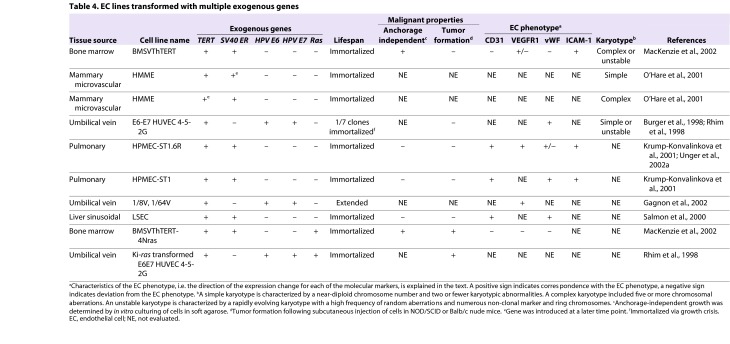
EC lines transformed with multiple exogenous genes

Overexpression of oncogenic N-Ras mediated tumorigenic conversion of immortalized BMECs (MacKenzie et al., 2002). Upon subcutaneous injection into immunocompromised mice, BMECs that co-expressed hTERT, SV40 T antigen and N-Ras generated large, rapidly growing tumors that were organized into lumen-like structures and in some mice metastasized to lymph nodes (MacKenzie et al., 2002). When injected via the tail vein, the ECs lodged within the lungs, where they formed tumors that caused morbidity within 3 weeks of implantation. Similarly, human umbilical vein endothelial cells (HUVECs) transfected with HPV E6 and E7 oncogenes and immortalized via spontaneous activation of telomerase were vulnerable to tumorigenic conversion by transfection with activated K-ras (Rhim et al., 1998). The demonstration of K-ras mutations in angiosarcoma underscores the relevance of these models to human vascular neoplasias (Boivin-Angèle et al., 2000; Hong et al., 2000).

## EC hybrid and vascular tumor-derived cell lines

In addition to genetically engineered ECs, immortalized EC lines have been generated by fusion of HUVECs with human tumor cell lines, such as the adenocarcinoma A549 cell line (Table 5) (Edgell et al., 1983; Hohenwarter et al., 1992a; Unger et al., 2002b). Hybrid endothelial tumor cell lines have unlimited replicative potential and retain some, but not all, EC characteristics (Unger et al., 2002a). In addition, because the hybrid EC A549 cell line displays karyotypic changes that have also been identified in A549 cells, it does not seem an ideal representation of the molecular biology of endothelial tumor cells (Edgell et al., 1983).

**Table 5.**
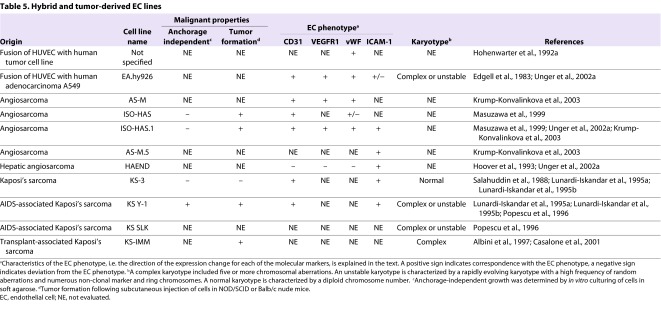
Hybrid and tumor-derived EC lines

Authentic EC lines isolated directly from vascular tumors are clearly valuable for studies of the molecular biology of the disease. Cell lines established by *ex vivo* culture of human tumor tissues include the angiosarcoma-derived cell lines ISO-HAS, AS-M and HAEND (Hoover et al., 1993; Masuzawa et al., 1999; Unger et al., 2002a; Krump-Konvalinkova et al., 2003). Aberrant p53 expression, a lack of telomerase activity and an ability to form tumors *in vivo* (evaluated in ISO-HAS and ISO-HAS.1) are some of the characteristics that angiosarcoma-derived clonal EC lines have in common with primary angiosarcomas. At least four KS-derived EC lines have been established from AIDS- and non-AIDS-associated KS patients (Table 5) (Salahuddin et al., 1988; Lunardi-Iskandar et al., 1995b; Popescu et al., 1996; Albini et al., 1997; Casalone et al., 2001). Most of the reported EC-tumor-derived cell lines retained typical endothelial characteristics, including expression of CD31, VEGFR1, vWF and I-CAM. One exception was the HAEND cell line, which suppressed expression of all of the defining EC markers that were examined in two separate reports (Hoover et al., 1993; Unger et al., 2002b).

It is also important to mention that two presumptive EC lines, ECV304 and TEC61, which were broadly used until ∼2000, were shown by DNA profile analysis to be misidentified cell lines (MacLeod et al., 1999). ECV304 was originally reported as an immortal EC line derived from spontaneous transformation of HUVECs, whereas TEC61 was established from thyroid endothelium. However, ECV304 was subsequently found to be genetically identical to the T24 bladder carcinoma cell line and TEC61 was identified as the human choriocarcinoma cell line, JEG3 (http://www.atcc.org/en/Products/Cells_and_Microorganisms/Cell_Lines/Misidentified_Cell_Lines.aspx). These discoveries highlight the importance of cell line authentication and characterization of endothelial phenotypes prior to the utilization of presumptive tumor-derived cell lines in research and drug development. It is also of consideration that tumor-derived cell lines represent end-stage disease, and therefore will be most valuable when used in conjunction with well-characterized EC lines that have been genetically manipulated to represent earlier stages of EC transformation.

## Animal models of vascular neoplasias

In addition to cell lines, animal models are required for preclinical testing of novel therapeutics. Models that have been established for *in vivo* study of vascular neoplasias include mice and non-human primates treated with chemical carcinogens or infected with KSHV. Whereas infection with purified KSHV provides a relevant model of KS (Dittmer et al., 1999; Mutlu et al., 2007; Chang et al., 2009), the high incidence of Ras mutations in chemically induced murine vascular neoplasia is consistent with human angiosarcoma (Hong et al., 1980; Sato et al., 1986). Other murine models include mice with a combined knock out of *TP53* and the gene encoding p18^INK4c^, which results in the development of angiosarcomas as well as other cancers of soft tissue (Harvey et al., 1993; Zindy et al., 2003). Interestingly, *Notch1* knockout mice were also found to develop hepatic angiosarcoma with a very high penetrance (Dill et al., 2012). However, to date, Notch1 defects have not been demonstrated in human angiosarcoma.

Clinical trials of novel therapeutics are usually preceded by *in vivo* tests using patient-derived cell lines or genetically engineered cell lines xenografted in animal models. Tumors that form in mice xenoengrafted with human ECs co-expressing viral antigens that inactivate tumor suppressors p53 and pRb, together with exogenous expression of hTERT and oncogenic Ras, could be used to model angiosarcoma or malignant hemangioendothelioma (Table 4) (Rhim et al., 1998; MacKenzie et al., 2002). By contrast, cavernous endothelial tumors established by infection of neonatal mice with polyoma virus middle T antigen, which induces constitutive tyrosine kinase activity, might be a more relevant model of infantile hemangioma (Bautch et al., 1987). A benign neoplasia resembling hemangioma was also modeled in mice xenografted with HUVECs expressing polyoma virus middle T antigen. The xenografted cells formed tumors that regressed after 3 weeks, mirroring the typical course of hemangioma (Primo et al., 2000; Schaffhausen and Roberts, 2009).

Engraftment of patient-derived cells in immunocompromised mice is a clinically relevant alternative to genetically engineered xenograft models. Similar to the polyoma middle T-antigen-driven xenograft tumors, patient-derived hemangioma progenitor cells were shown to form transient subcutaneous tumors when engrafted in immunocompromised mice (Khan et al., 2008; Xu et al., 2011). Xenograft tumors have also been established from canine angiosarcoma (Akhtar et al., 2004; Kodama et al., 2009). In addition to providing a useful model for preclinical testing of novel therapeutics, xenografts potentially provide a means for expansion of rare tumor material via serial transplantation (Khan et al., 2008).

## Perspectives and conclusions

Thus far, investigations of the molecular biology of EC-derived malignancies have been limited to molecular and cytogenetic case studies and investigations of specific oncogenes and tumor suppressors in small patient cohorts. The data accumulated to date implicate abnormalities in the VEGF pathway as a common event across the spectrum of benign and malignant vascular neoplasias (Fig. 1). Defects in the *TP53* and p16^INK4a^-RB tumor suppressor pathways feature in intermediate and aggressive malignancies, and are often associated with the development of chromosomal abnormalities. It is notable that, aside from aberrant VEGF signaling, there have been no reports of oncogenic changes that clearly distinguish EC neoplasias from other cancers, such as epithelial cancers. Hence, genetic models of EC neoplasias have largely relied on combinations that have also been shown to transform epithelial cells (Boehm and Hahn, 2005).

Studies involving the genetic manipulation of human ECs have confirmed that perturbation of *TP53* and p16^INK4a^-RB tumor suppressor pathways, together with the activation of a telomere maintenance mechanism, are crucial for EC immortalization. However, in contrast to carcinomas, which predominantly rely on telomerase for telomere maintenance, the limited literature available suggests that the activation of ALT might be relatively common in angiosarcoma. Nevertheless, ALT-immortalized EC lines are underrepresented among the currently available EC lines. In contrast to the well-documented approach to the reconstitution of telomerase by overexpression of hTERT, there are currently no known means of directly activating ALT in primary mammalian cells. However, studies that showed spontaneous activation of ALT in SV40-transformed epithelial and fibroblastic cell lines allude to an indirect means of establishing ALT-immortalized EC lines (Bryan et al., 1995).

The currently available genetic models might also be made more clinically relevant by substituting viral oncogenes with short hairpin RNA (shRNA) targeting the specific tumor suppressor genes implicated in EC malignancies, and by constitutive activation of VEGF signaling. Conditional deletion of tumor suppressor genes specifically within ECs would also provide a valuable alternative murine model of endothelial neoplasia. Additional refinement of genetic models will then be contingent upon further characterization of the specific molecular defects that underpin the initiation and progression of specific vascular neoplasias. Although hampered by sample numbers, genome-wide mapping and next-generation sequencing might be applied toward this goal. Information from broad-platform technologies such as these will be valuable for improving diagnosis, identifying therapeutic targets, and establishing new *in vitro* and *in vivo* models that accurately depict the molecular biology of specific EC-derived neoplasias. Such models will then provide an avenue for preclinical tests of new therapeutics and for ultimately improving outcomes for patients with these disorders.
